# Nutritional effects of the dietary inclusion of partially defatted *Hermetia illucens* larva meal in Muscovy duck

**DOI:** 10.1186/s40104-019-0344-7

**Published:** 2019-05-10

**Authors:** Marta Gariglio, Sihem Dabbou, Ilaria Biasato, Maria Teresa Capucchio, Elena Colombino, Fuensanta Hernández, Josefa Madrid, Silvia Martínez, Francesco Gai, Christian Caimi, Sara Bellezza Oddon, Marco Meneguz, Angela Trocino, Riccardo Vincenzi, Laura Gasco, Achille Schiavone

**Affiliations:** 1 Department of Veterinary Sciences, University of Turin, largo Paolo Braccini 2, Turin, Grugliasco 10095 Italy; 2 Department of Agricultural, Forest and Food Sciences, University of Turin, largo Paolo Braccini 2, Turin, Grugliasco 10095 Italy; 3 Department of Animal Production, University of Murcia, Campus de Espinardo, 30071 Murcia, Spain; 4 Institute of Science of Food Production, National Research Council, largo Paolo Braccini 2, Turin, Grugliasco 10095 Italy; 5 Department of Comparative Biomedicine and Food Science, University of Padua, viale dell’Università 16, Padua, Legnaro 35020 Italy; 6 A.I.A. Agricola Italiana Alimentare S.p.A, via Val Pantena 18G, 37142 Verona, Italy; 7 Institute of Interdisciplinary Research on Sustainability, University of Turin, via Accademia Albertina 13, 10100 Turin, Italy

**Keywords:** Black soldier fly, Digestibility, Ducks, Histopathology, Insect, Performance

## Abstract

**Background:**

The present work is aimed at evaluating the effect of different inclusion levels of a partially defatted black soldier fly (*Hermetia illucens*, L.; HI) larva meal for ducks. A total of 192 female 3-day-old Muscovy ducklings (*Cairina moschata domestica*, Canedins R71 L White, Grimaud Freres Selection, France) were divided into 4 groups, assigned 4 different dietary treatments (6 replicates/treatment and 8 birds/replicate) and reared from 3 to 50 days of age. HI larva meal was included at increasing levels (0, 3%, 6% and 9%, HI0, HI3, HI6 and HI9, respectively) in isonitrogenous and isoenergetic diets formulated for 3 feeding phases: starter (3–17 days of age), grower (18–38 days of age) and finisher (39–50 days of age). The growth performance and apparent total tract digestibility were evaluated during the trial using titanium dioxide as an inert marker (0.3% of inclusion). At 51 days of age, two birds per pen were slaughtered and histomorphological investigations were performed.

**Results:**

The live weight and average daily gain showed a quadratic response to increasing HI meal in the grower period (minimum corresponding to the HI6 group). No effects of dietary inclusion levels were observed for the daily feed intake or feed conversion ratio. The apparent dry matter and organic matter digestibility were not affected by the dietary treatment. A linear decrease was observed for the crude protein apparent digestibility in the starter period (minimum for the HI9 groups). The ether extract apparent digestibility increased linearly during the grower and finisher periods (minimum for the HI0 group). The morphometric indices were not influenced by the dietary treatments.

**Conclusions:**

The inclusion of up to 9% of HI partially defatted larva meal in the diet of ducks did not cause any effect on growth performance, as well as the apparent digestibility. Moreover, dietary HI inclusion preserved the physiological intestinal development.

## Introduction

With an increase of 1.8% per year estimated until 2050, poultry production is one of the fastest growing sectors [1]. The production of duck meat has undergone a great expansion in recent years, passing from 2.23 million tonnes in 1996 to over 4.5 million tonnes in 2016 [2]. Duck production plays an important role in Italy, with 3.27 thousand tonnes being produced in 2017 [3]. Duck farming has a great potential, since ducks are among the fastest producers of animal proteins and may contribute to the future nutritional needs of the growing world population [4].

The reduced availability of natural resources and the environmental impact of vegetable production require the research of alternative forms of protein for animal production. Considering the feeding habits of birds, including poultry (chicken, duck, turkey and geese), insects can represent a valuable alternative to the common protein sources, thanks to their nutritional characteristics [5]. The use of insects in feeds leads to advantages from both an environmental and nutritional point of view. Compared to conventional vegetable protein sources, insects rearing requires very little soil and water, and their low emission of greenhouse gases and ammonia implies an overall low environmental impact. A study conducted by an EU initiative PROteINSECT estimated that 1 ha of land can produce up to 150 tons of insect protein per year. Instead, 1 ha of land can produce less than 1 ton of soybean protein [6]. Moreover, insects can be reared on organic waste (such as fruit and vegetable) and can converting them into nutrients of high biological value, thanks to their high feed conversion efficiency [7, 8]. Researchers have recently been evaluating the use of insects in animal feeding, and they have highlighted that insects could be used as a partial or total substitute for the currently used protein sources. The most promising species are *Hermetia illucens* (HI) [9–12], *Musca domestica* [13, 14] and *Tenebrio molitor* (TM) [15–18]. Thanks to the favourable nutrient composition of HI, in terms of protein content (37–63%), amino acid profile, fat content (7–39%) and other macro- and micronutrients, it could be considered a valuable alternative to common protein sources, such as soybean meal (SBM) [19]. The effects of the inclusion of HI larva meal and oil on growth performance have been investigated in many studies [10–12, 20–22], with conflicting results being obtained. Only a few works are available regarding the digestibility of HI meal in poultry [9, 10, 22–24] and these show extremely variable results in terms of dry matter (DM), crude protein (CP) and ether extract (EE). In these studies, nutrient digestibility was frequently influenced, according to the authors’ interpretation, by the chitin content of the diets, which can negatively affect digestibility. Despite the limited availability of studies, the evaluation of the gut histomorphometry represents a relevant research topic, since it could affect the growth performance and the digestibility of a diet [25], depending on the protein source and level of the diet [26, 27]. A recent study by Cutrignelli et al. [22] has found that the inclusion of HI larva meal in laying hen diets significantly affects the gut histomorphometry, resulting in a lower villi-to-crypt ratio than SBM group. The above-mentioned studies have shown a wide variability and more research is required to obtain a better understanding of the effect of the inclusion of HI in poultry diets.

To date, no research has investigated the possibility of including insect meal in duck diet. Therefore, the aim of the study was to evaluate the growth performance, digestibility and intestinal morphology of female broiler ducks (*Cairina moschata domestica*) fed diets including HI meal.

## Materials and methods

### Birds and husbandry

The present trial was performed at the poultry facility of the University of Turin (Italy). The experimental protocol (Prot. No. 380576, 4^th^ December 2017) was approved by the Bioethical Committee of the University of Turin (Italy). The poultry house was 7 m wide × 50 m long × 7 m high, and was equipped with a waterproof floor and walls, completely covered by tiles and had an automatic ventilation system. A total of 192, 3-day-old, females Muscovy ducks (Canedins R71 L White, Grimaud Freres Selection, France) were housed in 24 pens and randomly allotted 4 dietary treatments, each group consisting of 6 pens as replicates with 8 birds per pen (average live weight (LW): 71.32 ± 2.95 g). Each pen was 1.20 m wide × 2.20 m long and was covered with rice hulls as litter. During the first 3 weeks, the animals were heated by infrared lamps. The lighting schedule was 23 h light : 1 h darkness until day 3 of the trial, and thereafter the dark period was gradually increased to 6 h and maintained constant until slaughtering. The environmental parameters were monitored daily during the whole period of the trial.

### Diets

Four dietary treatments were obtained in which increasing levels of a partially defatted HI larva meal (Hermetia Deutschland GmbH & Co KG, Baruth/Mark, Germany) were included. In order to assess the effect of HI meal inclusion in the diets, HI meal was included as a substitute to gluten meal, a commonly used raw material in commercial duck feeding, which nutritional value is comparable to HI meal.

The control group (HI0) was fed a diet without insect meal (9% inclusion of corn gluten meal); 3%, 6% and 9% of the gluten meal was substituted with HI larva meals in the HI3, HI6, and HI9 diets, respectively (Table 1).

**Table 1:** Ingredients (g/kg as fed) and nutrient composition (%) of the experimental diets^a^

Ingredients	Starter period (days 3 to 17)				Grower period (days 18 to 38)				Finisher period (days 39 to 50)			
	HI0	HI3	HI6	HI9	HI0	HI3	HI6	HI9	HI0	HI3	HI6	HI9
Corn meal	600.0	600.0	600.0	600.0	638.0	638.0	638.0	638.0	670.0	670.0	670.0	670.0
Soybean meal	212.0	212.0	212.0	212.0	160.0	160.0	160.0	160.0	100.0	100.0	100.0	100.0
HI larva meal	0.0	30.0	60.0	90.0	0.0	30.0	60.0	90.0	0.0	30.0	60.0	90.0
Bran	42.5	42.5	42.5	42.5	36.3	36.3	36.3	36.3	66.2	66.2	66.2	66.2
Corn gluten meal	90.0	60.0	30.0	0.0	90.0	60.0	30.0	0.0	90.0	60.0	30.0	0.0
Soybean oil	16.5	16.5	16.5	16.5	28.5	28.5	28.5	28.5	34.5	34.5	34.5	34.5
Dicalcium phosphate	10.0	10.0	10.0	10.0	13.0	13.0	13.0	13.0	4.0	4.0	4.0	4.0
Calcium carbonate	8.0	8.0	8.0	8.0	14.0	14.0	14.0	14.0	17.4	17.4	17.4	17.4
Sodium chloride	2.5	2.5	2.5	2.5	2.5	2.5	2.5	2.5	2.5	2.5	2.5	2.5
Sodium bicarbonate	2.0	2.0	2.0	2.0	2.0	2.0	2.0	2.0	2.0	2.0	2.0	2.0
*DL*-methionine	2.5	2.5	2.6	2.8	1.7	1.8	1.9	2.2	0.3	0.4	0.5	0.8
*L*-lysine	3.9	3.9	3.8	3.6	3.9	3.8	3.7	3.4	3.0	2.9	2.8	2.5
Mineral-vitamin premix^b^	5.0	5.0	5.0	5.0	5.0	5.0	5.0	5.0	5.0	5.0	5.0	5.0
Choline chloride	0.1	0.1	0.1	0.1	0.1	0.1	0.1	0.1	0.1	0.1	0.1	0.1
Optifos 250 bro	1.0	1.0	1.0	1.0	1.0	1.0	1.0	1.0	1.0	1.0	1.0	1.0
Avizyme 1500×	1.0	1.0	1.0	1.0	1.0	1.0	1.0	1.0	1.0	1.0	1.0	1.0
Titanium dioxide	3.0	3.0	3.0	3.0	3.0	3.0	3.0	3.0	3.0	3.0	3.0	3.0
Total	1,000	1,000	1,000	1,000	1,000	1,000	1,000	1,000	1,000	1,000	1,000	1,000
AMEn^c^, kcal/kg	2,897	2,892	2,888	2,884	2,994	2,990	2,986	2,981	3,052	3,048	3,044	3,040
Nutrient composition^d^												
DM, %	89.3	89.8	90.1	89.7	88.8	89.2	89.1	89.1	88.7	89.0	89.3	88.9
CP, % DM	25.1	24.7	25.2	24.8	23.0	22.5	22.5	22.4	20.2	20.2	20.1	20.1
EE, % DM	4.7	4.8	4.9	5.1	6.2	6.2	6.4	6.6	7.3	7.4	7.6	7.7
NDF, % DM	12.7	12.3	12.7	12.4	12.9	13.1	12.6	12.7	12.7	13.1	12.7	12.9
ADF, % DM	3.4	3.5	3.7	3.3	3.5	3.5	3.7	3.5	3.4	3.5	3.5	3.4
Ash, % DM	5.6	6.0	5.6	5.8	7.8	7.5	7.5	8.0	6.5	6.4	6.9	6.7
Fatty acid composition, % of total FAME												
SFA	19.0	20.7	23.9	26.9	17.7	20.2	22.5	25.5	17.3	19.4	22.2	23.7
MUFA	27.6	26.1	25.5	24.6	25.8	24.9	24.2	23.5	24.9	24.8	23.8	23.8
PUFA	52.8	52.6	49.9	47.9	55.9	54.4	52.7	50.5	57.2	55.3	53.5	51.8
PUFA/SFA	2.78	2.54	2.08	1.78	3.15	2.69	2.34	1.98	3.31	2.85	2.40	2.18
∑*n*-3	3.3	3.4	3.4	3.3	4.1	4.2	4.1	4.1	4.5	4.5	5.3	5.0
∑*n*-6	49.1	48.9	46.1	44.3	51.6	49.9	48.2	46.1	52.3	50.4	47.9	46.4
∑*n*-6/∑*n*-3	14.88	14.38	13.56	13.42	12.58	11.88	11.76	11.24	11.62	11.20	9.04	9.28

*HI Hermetia illucens*, *AMEn* apparent metabolisable energy, *DM* dry matter, *CP* crude protein, *EE* ether extract, *NDF* neutral detergent fiber, *ADF* acid detergent fiber, *FAME* fatty acid methyl esters, *SFA* saturated fatty acids, *MUFA* monounsaturated fatty acids, *PUFA* polyunsaturated fatty acids

^a^Four dietary treatments: HI0 = control; HI3 = 3% inclusion level of *Hermetia illucens*; HI6 = 6% inclusion level of *Hermetia illucens*; HI9 = 9% inclusion level of *Hermetia illucens*

^b^Mineral-vitamin premix: vitamin A (retinyl acetate), 12,500 IU; vitamin D_3_ (cholecalciferol), 3,500 IU; vitamin E (*DL*-a-tocopheryl acetate), 40 mg; vitamin K (menadione sodium bisulfite), 2.0 mg biotin, 0.20 mg; thiamine, 2.0 mg; riboflavin, 6.0 mg; pantothenate, 15.21 mg; niacin, 40.0 mg; choline, 750.0 mg pyridoxine, 4.0 mg; folic acid, 0.75 mg; vitamin B_12_, 0.03 mg; Mn, 70 mg; Zn, 62.15 mg; Fe, 50.0 mg; Cu, 7.0 mg; I, 0.25 mg; Se, 0.25 mg

^c^Calculated according to Schiavone et al. [23] for HI meal and INRA [28] for the other ingredients

^d^The chemical analyses were carried out on three replicates of each feed sample

The diets were isonitrogenous and isoenergetic and were formulated using the apparent metabolisable energy (AMEn) values for a partially defatted HI, which had been calculated by Schiavone et al. [23] and according to INRA for the other ingredients [28] both for broiler chickens. The essential amino acids requirements were calculated with matrix value for digestible amino acids according to Schiavone et al. [23] for HI meal and Pingel et al. [29] for the other ingredients, for chickens and ducks, respectively. Feed and water were provided *ad libitum* throughout the trial. A 3-phase feeding program was applied: a starter diet (days 3 to 17), a grower diet (days 18 to 38) and a finisher diet (days 39 to 51).

### Chemical analysis of the HI meal and experimental diets

The experimental diets were ground to pass through a 0.5-mm sieve and stored in airtight plastic containers. HI larva meal and the experimental diets were analysed for DM (AOAC, method number #934.01), ash (AOAC, method number #942.05), CP (AOAC, method number #984.13), neutral detergent fiber (NDF; AOAC, method number #2002.04) and acid detergent fiber (ADF; AOAC method number #973.18) [30]. The EE (AOAC, method number #2003.05) was determined according to International AOAC [31].

The chitin content of HI meal was determined according to Finke et al. [32] using ADF adjusted for its nitrogen content.

In order to perform the amino acids determination in HI meal, samples were prepared using a 22-h hydrolysis step in 6 mol/L HCl at 112 °C under a nitrogen atmosphere. Performic acid oxidation occurred prior to acid hydrolysis for methionine and cystine. The amino acids in hydrolysate was determined by means of HPLC after postcolumn derivatization, according to the procedure described by Madrid et al. [33]. Tryptophan was not determined. The lipid extraction and fatty acids profiling of the experimental diets [34–36] were carried out at the laboratory of the Department of Animal Medicine, Production and Health, University of Padua, Legnaro, Italy according to the method of Christie [37]. All the analysis were performed in triplicate (Table 1).

The chemical composition of the HI meal was the following: DM, 92.41 g/kg; CP, 56.71% DM; EE, 10.70% DM; ash, 16.38% DM; chitin, 6.43% DM; *DL*-methionine, 0.63% DM and *L*-lysine, 1.89% DM.

### Growth performance

Birds were individually labeled with a wing mark and weighed at their arrival. Mortality and clinical signs of illness were monitored daily throughout the trial. The live weight (LW) of the animals was recorded at an individual level at the beginning and at the end of each feeding phase (3, 17, 38 and 50 days of age), and the feeds were removed 2 h before the birds were weighed. The average daily gain (ADG) and average daily feed intake (DFI) were recorded at a pen level at the end of each growth period.

The feed conversion ratio (FCR) was calculated for each growth period and for the overall experimental period. All the measurements were made using electronic scales (Sartorius-Signum®, Bovenden, Germany).

### Digestibility trial

The digestibility trial was performed at the end of each feeding phase using titanium dioxide (TiO_2_, 0.3 g/kg) as an indigestible marker in each experimental diet (Table 1), in order to evaluate the apparent total tract digestibility coefficients (ATTDC). The Kaczmarek et al. [38] method was used to collect the excreta, with slight modifications, as reported by Dabbou et al. [39]. Briefly, all the birds were removed from each pen and housed in wire-mesh cages (*n* = 6 replicates) for approximately 1 h/d for four consecutive days to collect fresh excreta samples.

After collection, the excreta samples, from which the feathers and litter had been removed, were immediately frozen at − 20 °C. At the end of each collection period, the excreta were pooled, lyophilized, grounded and stored at 4 °C. All the analyses were carried out on two replicates for each sample. The ATTDC was evaluated for DM, CP, EE and OM. The uric acid (UA) content in the excreta samples was determined spectrophotometrically (UNICAN UV–Vis Spectrometry, Helios Gamma, the United Kingdom) according to the Marquardt method [40]. The nitrogen contained in uric acid is the 33.33%. The CP amount in the excreta (CP *corrected*) was calculated using the excreta CP, corrected for UA as follows:

$$ \mathrm{CP}\  corrected=\left(\mathrm{total}\ \mathrm{nitrogen}-\mathrm{UA}\hbox{-} \mathrm{nitrogen}\right)\times 6.25. $$

The TiO_2_ content was measured on a UV spectrophotometer (UNICAN UV–vis Spectrometry, Helios Gamma, the United Kingdom) following the Myers et al. [41] method .

The ATTDC of the dietary nutrients was calculated using the following method [42]:

$$ ATTDC\ {X}_{diet}=\left[\frac{\left( Total\ X\  ingested- total\ X\  excreted\right)}{total\ X\  ingested}\right] $$

$$ Digestibility=\left[\frac{\left(\%{X}_{diet}/\%{TiO}_{diet}\right)-\left(\%{X}_{excreta}/\%{TiO}_{excreta}\right)}{\left(\%{X}_{diet}/\%{TiO}_{diet}\right)}\right] $$

where *X* represents DM, CP, EE or OM.

### Slaughtering procedures

At 50 days of age, the final LW of the birds was recorded individually and 12 ducks per diet (two birds per pen) were chosen on the basis of the LW pen average and identified by means of a shank ring. Then the feed was removed and, after 12 h of fasting (at 51 days of age), the selected animals were transferred to a commercial abattoir and slaughtered by electrical stunning and bleeding, according to the standard EU regulations. The plucked and eviscerated carcasses were obtained, the head, neck and feet were removed.

### Histomorphological investigations

Gut segments (approximately 5 cm in length, 12 animals per each group) of the duodenum, jejunum and ileum were sampled during slaughtering and flushed with 0.9% saline to remove all the contents. The collected intestine segments were the loop of the duodenum, the tract before Meckel’s diverticulum (jejunum) and the tract before the ileocolic junction (ileum). The gut samples were fixed in a 10% buffered formalin solution, routinely embedded in paraffin wax blocks, sectioned at a 5-μm thickness, mounted on glass slides and stained with Haematoxylin & Eosin (HE) for morphometric analysis. The evaluated morphometric indices were as follows: villus height (Vh, from the tip of the villus to the crypt), crypt depth (Cd, from the base of the villus to the submucosa) and the villus height-to-crypt depth (Vh/Cd) ratio [27]. Morphometric analyses were performed on 10 well-oriented and intact villi and 10 crypts chosen from the duodenum, jejunum and ileum [26].

### Statistical analysis

The statistical analyses were performed using the SPSS software package (version 21 for Windows, SPSS Inc., Chicago, IL, USA). The mortality rate was analysed by means of a Chi-square test, using the HI0 group as the reference. Shapiro-Wilk’s test established normality or non-normality of distribution. The assumption of equal variances was assessed by means of Levene’s homogeneity of variance test. The experimental unit was the pen for growth performance and digestibility, while the duck was used for the intestinal morphology. The collected data were tested by means of one-way ANOVA. Polynomial contrasts were used to test the linear and quadratic responses to increases in the HI inclusion level in the diet. Intestinal morphometric indices were analysed by fitting a general linear mixed model (GLMM). GLMM allowed the morphometric indices (Vh, Cd and Vh/Cd, separately) to depend on three fixed factors (diet, intestinal segment and interaction between diet and intestinal segment). Animal was included as a random effect to account for repeated measurements in the same duck. The interactions between the levels of the fixed factors were evaluated by means of pairwise comparisons.

Differences among treatments were considered statistically significant when the *P* values ≤0.05.

## Results

### Growth performance

The cumulative mortality rates of the HI0 (4.16%), HI3 (2.08%), HI6 (2.08%) and HI9 (2.08%) groups were not influenced by the dietary treatments (*P* > 0.05). The growth performances of the broiler ducks are summarized in Table 2. Overall LW was not influenced by the dietary treatments (*P* > 0.05), except at 38 days of age, when a quadratic response was observed in LW for increasing HI meal levels with a minimum being observed for the HI6 group (*P* < 0.05).

**Table 2:** Effect of the dietary HI larva meal inclusion on the growth performance of female ducks (*n* = 6)

Items	Age	Dietary treatments^a^				SEM	*P*-value	
		HI0	HI3	HI6	HI9		Linear	Quadratic
LW, g	3 d	70.70	70.41	72.65	71.51	0.60	0.405	0.733
	17 d	575.44	567.31	572.56	575.74	4.76	0.893	0.582
	38 d	1906.79	1861.96	1797.10	1900.12	14.18	0.426	0.005
	50 d	2540.57	2511.14	2456.14	2554.84	20.13	0.946	0.123
ADG, g/d	3–17 d	36.05	35.49	35.71	36.02	0.32	0.974	0.529
	18–38 d	63.40	61.65	58.31	63.07	0.69	0.417	0.011
	39–50 d	52.81	54.10	54.92	54.56	1.14	0.582	0.738
	3–50 d	52.55	51.93	50.71	52.84	0.43	0.926	0.125
DFI, g/d	3–17 d	53.69	53.45	52.16	51.85	0.59	0.226	0.979
	18–38 d	142.03	139.06	136.99	139.92	1.28	0.481	0.273
	39–50 d	167.48	170.82	160.32	171.80	2.83	0.924	0.485
	3–50 d	120.58	121.32	117.58	121.48	1.20	0.927	0.530
FCR, g/g	3–17 d	1.49	1.51	1.46	1.44	0.01	0.099	0.489
	18–38 d	2.24	2.26	2.35	2.22	0.03	0.913	0.159
	39–50 d	3.17	3.17	2.93	3.18	0.051	0.639	0.220
	3–50 d	2.29	2.34	2.32	2.30	0.019	0.925	0.406

*HI Hermetia illucens*, *SEM* standard error of the mean, *LW* live weight, *ADG* average daily gain, *DFI* daily feed intake, *FCR* feed conversion ratio

^a^Four dietary treatments: HI0 = control; HI3 = 3% inclusion level of *Hermetia illucens*; HI6 = 6% inclusion level of *Hermetia illucens*; HI9 = 9% inclusion level of *Hermetia illucens*

ADG was not affected by the dietary treatments (*P* > 0.05), with the exception of the HI6 group in the second period (18–38 days of age), where the ADG showed a quadratic response (*P* < 0.05). DFI and FCR were not affected by the dietary treatment nor in the different feeding phases or over the whole experimental trial (*P* > 0.05).

### Digestibility trial

The apparent digestibility coefficients are reported in Table 3. DM digestibility was not affected by the dietary treatment throughout the trial, as well as the OM (*P* > 0.05). In the starter period (3–17 days of age) the CP digestibility decreased linearly with a minimum corresponding to the HI9 groups (*P* < 0.05) (–4.11% compared to HI0, respectively), whereas the EE digestibility decreased linearly with the inclusion of HI in the diets (*P* < 0.05).

**Table 3:** Effect of the dietary inclusion of HI larva meal on the nutrients apparent digestibility of Muscovy ducks (*n* = 6)

Age	Apparent digestibility	Dietary treatments^a^				SEM	*P* -value	
		HI0	HI3	HI6	HI9		Linear	Quadratic
3–17 d	DM	0.960	0.960	0.960	0.953	0.002	0.174	0.315
	CP	0.852	0.872	0.828	0.817	0.007	0.010	0.195
	EE	0.945	0.967	0.963	0.962	0.002	0.003	0.085
	OM	0.963	0.963	0.966	0.962	0.001	0.853	0.453
18–38 d	DM	0.953	0.956	0.962	0.960	0.002	0.086	0.511
	CP	0.800	0.802	0.802	0.828	0.005	0.085	0.269
	EE	0.958	0.966	0.968	0.977	0.002	< 0.001	0.891
	OM	0.958	0.964	0.967	0.963	0.001	0.215	0.168
39–50 d	DM	0.943	0.948	0.952	0.953	0.002	0.099	0.703
	CP	0.733	0.682	0.715	0.718	0.012	0.913	0.259
	EE	0.953	0.958	0.965	0.983	0.003	< 0.001	0.092
	OM	0.950	0.953	0.958	0.958	0.002	0.072	0.642

*HI Hermetia illucens*, *SEM* standard error of the mean, *DM* dry matter, *CP* crude protein, *EE* ether extract

^a^Four dietary treatments: HI0 = control; HI3 = 3% inclusion level of *Hermetia illucens*; HI6 = 6% inclusion level of *Hermetia illucens*; HI9 = 9% inclusion level of *Hermetia illucens*

In the periods from 18 to 38 days of age and from 39 to 50 days of age, the EE digestibility showed a linear increase, with a maximum corresponding to the HI9 group (*P* < 0.001) (+ 1.94% and + 3.05% compared to HI0 in the grower and finisher periods, respectively). However, CP digestibility was not affected by the dietary treatments in the grower and finisher periods (*P* > 0.05).

### Histomorphological investigations

The effects of the diet, gut segment and interaction between the diet and gut segment on the gut morphometric indices of the ducks are summarized in Table 4. The intestinal segment significantly affected Vh, Cd and Vh/Cd (*P* < 0.001). On the other hand, no influence of diet or interaction between the diet and intestinal segment (*P* > 0.05) were observed on the morphometric indices. The duodenum showed higher Vh and Cd values (*P* < 0.05 and *P* < 0.01, respectively) than the ileum, and the morphometric indices were also greater (*P* < 0.05 and *P* < 0.01, respectively) in the jejunum than in the ileum. The duodenum also showed a greater Vh/Cd (*P* < 0.05) than the other gut segments.

**Table 4:** Intestinal morphometric indices in the ducks in relation to diet and intestinal segment (*n* = 12, end of the trial)

Index	Diet (D)^d^				Intestinal segment (IS)^e^			SEM		*P*-value		
	HI0	HI3	HI6	HI9	DU	JE	IL	D	IS	D	IS	D × IS
Vh, mm	1.55	1.51	1.52	1.63	2.12^a^	1.41^b^	1.14^c^	0.06	0.06	0.442	< 0.001	0.508
Cd, mm	0.16	0.14	0.15	0.15	0.18^a^	0.15^b^	0.13^c^	0.01	0.01	0.346	< 0.001	0.782
Vh/Cd	9.80	11.06	10.67	10.83	12.62^a^	9.67^b^	9.48^b^	0.60	0.45	0.469	< 0.001	0.966

*Vh* villus height, *Cd* crypt depth, *Vh/Cd* villus height-to-crypt depth ratio

The means with different superscript letters (^a, b, c^) within the same row per fixed effect (i.e. diet, intestinal segment) differ significantly (*P* < 0.05)

^d^Four dietary treatments: HI0 = control; HI3 = 3% inclusion level of *Hermetia illucens*; HI6 = 6% inclusion level of *Hermetia illucens*; HI9 = 9% inclusion level of *Hermetia illucens*

^e^Three intestinal segments: *DU* duodenum, *JE* jejunum, *IL* ileum

## Discussion

### Growth performance

Currently, no literature is available regarding the use of insect meals in duck feeding. For this reason, all the comparisons with literature data referred to other poultry species fed with HI meals and other insect meals.

The final LW of the birds was in line with the weight reported by Pingel et al. [29]. The results showed that HI meal could be a valuable alternative to corn gluten meal, and HI meal can be included in duck diets by as much as 9% without any negative effects on the final LW, ADG, DFI and FCR of the animals. Despite the lower LW and ADG of HI6 birds in the grower period (18–38 days of age), DFI and FCR were not influenced by the dietary treatment, and the final LW of the HI6 group was in line with the weight of the other treatments. Similarly, Cullere et al. [10] did not observe any differences in the final LW of broiler quails (*Coturnix coturnix japonica*) fed two different diets at 10% and 15% of inclusion levels of HI meal (in substitution of SBM protein and oil). Our results also agree with what Bovera et al. [24] previously reported for laying hens fed 25% and 50% HI in substitution of SBM (73 g/kg and 146 g/kg of inclusion, respectively), thus showing that LW and DFI were not influenced by the dietary treatments. The inclusion of up to 10% of HI larva meal in the diet of broiler chickens influenced their final LW and also improved the DFI of chicks in the starter period [12]. As far as other insect species with potential interest as feeds are concerned, Adenjii [13] did not observe any dietary effects on the performance of broiler chickens when groundnut cake was substituted with housefly maggot (*M. domestica*) meal. Biasato et al. [16], Ramos-Elorduy et al. [43] and Bovera et al. [44] also reported that the inclusion of TM meal in broiler chicken diets (from 5% to 15% of inclusion) did not affect the final LW and DFI of the birds. On the other hand, the replacement of SBM with 25% and 50% of HI meal (100 and 190 g/kg of inclusion, respectively) and 25% TM meal (120 g/kg of inclusion) in Barbary partridge (*Alectoris barbara*) resulted in a higher LW than the control [45]. Finally, the results reported by Khan et al. [46], pertaining to broiler chicks fed with silkworm (*Bombyx mori*), housefly maggot and TM in substitution of SBM (7.8%, 8.0% and 8.1% of inclusion, respectively), pointed out a higher LW in insect-fed chicks than in the control. In this trial, the FCR was not affected by the dietary treatments, according to the results of Elwert et al. [47] and Cullere et al. [10] pertaining to broiler chickens and broiler quails, respectively, fed with increasing levels of defatted HI meal. On the contrary, an improved FCR was observed in Barbary partridge fed diets with 25% and 50% of substitution of SBM with HI and TM meal [45]. An improved FCR was also reported by Khan et al. [46] (silkworm, housefly maggot, TM) and Bovera et al. [44] (TM), who found that the FCR of broiler chickens was lower in chicks fed insect meal than the control diet with SBM.

The use of HI larva meal as a substitute to corn gluten meal results to be suitable in Muscovy ducks feeding. During the whole experimental period, the growth performances observed in the present study were not influenced by dietary inclusion levels of HI meal, as already observed by other researches in quails and laying hens [10, 24]. Furthermore, the absence of gut histomorphological alterations can also explain the growth performance results, as well as the slightly affected nutrient digestibility.

### Digestibility trial

The herein obtained results show that the inclusion of HI larva meal in Muscovy duck diets partially affected the apparent digestibility coefficients of the nutrients. Consistently with our results, Cullere et al. [10] did not report any differences in DM and OM total tract apparent digestibility in broiler quails as a result of the HI inclusion level in the diet. On the contrary, in laying hens fed 17% HI meal in the diet, the apparent ileal digestibility of DM was lower in the group fed HI meal than in the control [22]. Results reported by Bovera et al. [24] showed that the inclusion of HI meal by up to 7.3% in laying hen diets did not affect the apparent DM ileal digestibility, compared to the control with vegetable protein meal, whereas the inclusion of 14.6% reduced the apparent DM ileal digestibility. In another study, the use of TM in broiler chicken diets worsened the apparent ileal DM digestibility compared to the control with SBM [44].

In our trial, the CP and EE apparent digestibility in the starter period (3–17 days of age) showed an opposite linear trend, with a reduction in CP digestibility (up to − 3.5% in HI9, compared to the control) and an improvement in EE digestibility (+ 2.0 in HI9, compared to the control) following the increasing inclusion levels of HI meal in the diet. The lower CP digestibility for the HI6 and HI9 groups in the starter period could be related to the higher chitin content of the diet, because of the higher inclusion level of HI meal [48]. In fact, Cutrignelli et al. [22] and Bovera et al. [24] also observed a reduction in the apparent ileal CP digestibility of laying hens compared to the control diet with SBM, and explained this result as a consequence of the presence of chitin in the diet. Indeed, De Marco et al. [9] assumed that the chitin, the structural component of the exoskeleton of insects, can negatively affect the nutrient digestibility, resulting as an indigestible fiber for domestic poultry. However, no differences in CP total tract apparent digestibility were reported by Cullere et al. [10] for broiler quails, after a substitution of protein/fat sources with HI larva meal of up to 15% of inclusion. However, in our trial, the apparent CP digestibility was similar during the grower (18–38 days of age) and finisher (39–50 days of age) periods, thus suggesting an adaptation to the chitin levels in the diet. The studies conducted by Tabata et al. [49, 50] showed that the birds have acid chitinase genes in their genome. In particular, in poultry (such as ducks), the acid chitinase is expressed mainly at the level of the glandular stomach. The level of acid chitinase mRNA in stomach tissue is regulated by feeding behaviour, which was higher in omnivorous species than in herbivorous and carnivorous species [50]. Moreover, it could be speculated that the chitin level in the diet could influence the acid chitinase expression with an overall improvement in feed digestibility.

In our trial, the EE apparent digestibility was higher in HI groups than the control group. However, despite this positive result, the EE apparent digestibility was only 2.2% and 1.9% higher than the control in the starter and grower periods respectively (3–17 and 18–38 days of age) and 3.0% higher than the control in the finisher period (39–50 days of age). This result partially agrees with the results of Cullere et al. [10], who found that the EE total tract apparent digestibility in broiler quails fed with a 15% inclusion level of HI was higher than a 10% group, but similar to the control diet. On the other hand, Cutrignelli et al. [22] and Bovera et al. [24] found that the apparent ileal EE digestibility in laying hens was similar for HI meal- and SBM-fed birds. The linear increase in EE digestibility is not supported by the fatty acids profile, in particular by the polyunsaturated fatty acids (PUFA) content, that is usually related to an EE digestibility improvement [51]. Indeed, in the present study the dietary PUFA content, as well as the ratio polyunsaturated fatty acids/saturated fatty acids (PUFA/SFA ratio), decreased following the HI larva meal inclusion (Table 1). The highest EE digestibility at the maximum inclusion level (HI9 group) could be related to the overall amount of EE in the diets (+ 7.84%, + 6.06% and + 5.19% higher in HI9 group than HI0 group in starter, grower and finisher periods, respectively) [52].

As a whole, the absent or moderate effects on nutrient ATTDC had no impact on the ducks’ growth performances, without affecting final LW, ADG, DFI and FCR.

### Histomorphological investigations

Dietary HI meal inclusion did not affect the gut morphology of the ducks of our study. Since the rapid growth of chickens has been reported to strictly depend on the morphological and functional integrity of the digestive tract [25], it is reasonable to hypothesize that insect meal utilization does not negatively influence gut development and, as a consequence, animal performance. The greater mucosal development observed in the duodenum than in the other gut segments is also in agreement with the previous studies available on broilers [11, 14–17, 53, 54], thus suggesting that insect meal utilization leads to the preservation of the physiological intestinal morphology. Indeed, the duodenum is the intestinal tract that undergoes the fastest cell renewal, and is also the first gut segment to receive the physical, chemical and hormonal stimuli caused by the presence of the diet in the lumen [54]. The obtained results about the preservation of gut histomorphology in all dietary groups contributes to validate what has been previously discussed in terms of nutrient digestibility and growth performances.

## Conclusions

To the best of the authors’ knowledge, this is the first study that has evaluated the possibility of including insect meal in duck nutrition, and to have demonstrated how HI larva meal can be a valuable protein sources for ducks. Increasing inclusion levels of a partially defatted HI meal in Muscovy duck diets did not affect the growth performances of the birds, which showed similar LW, ADG, DFI and FCR to the control group fed with corn gluten meal, and only had weak effects on the apparent total tract digestibility during the first stages of growth. Moreover, these increasing levels did not affect the intestinal morphology or cause histopathological alterations. From this preliminary investigation, it appears that HI larva meal can be included in duck feeding at levels of up to 9% of the diet, with no negative effects on growth, digestibility or animal health. Furthermore, the obtained results help to expand the information available about the use of insects in poultry nutrition.

## Abbreviations

ADF: Acid detergent fibre
ADG: Average daily gain
AMEn: Apparent metabolisable energy
ATTDC: Apparent total tract digestibility coefficients
Cd: Crypt depth
CP: Crude protein
DFI: Average daily feed intake
DM: Dry matter
DU: Duodenum
EE: Ether extract
FAME: Fatty acid methyl esters
FCR: Feed conversion ratio
GLMM: General linear mixed model
HE: Haematoxylin & Eosin
HI: *Hermetia illucens*
IL: Ileum
JE: Jejunum
LW: Live weight
MUFA: Monounsaturated fatty acids
NDF: Neutral detergent fiber
OM: Organic matter
PAS: Periodic acid-Schiff
PUFA: Polyunsaturated fatty acids
SB: Sudan Black
SBM: Soybean meal
SEM: Standard error of the mean
SFA: Saturated fatty acids
TM: *Tenebrio molitor*
Vh: Villus height
Vh/Cd: Villus height-to-crypt depth ratio
